# Psychometric evaluation of the PROMIS^®^ Medication Adherence Scale among patients prescribed oral anticancer medication for multiple myeloma

**DOI:** 10.1186/s41687-026-01204-z

**Published:** 2026-09-19

**Authors:** Sarah M. Belcher, Paul Scott, Susan M. Sereika, Catherine Bender, Jacqueline Dunbar-Jacob, Margaret Q. Rosenzweig, Benyam Muluneh, Lindsay M. Sabik, Valire Copeland, Mounzer Agha, Abigail A. Lustyik, Emily S. He, Corrine Bozich, Grace Booze, Petra Duran Basso, J. Devin Peipert

**Affiliations:** 1 School of Nursing, University of Pittsburgh, Pittsburgh, USA; 2 UPMC Hillman Cancer Center, Pittsburgh, USA; 3 Present Address: College of Nursing, The Ohio State University, Columbus, Ohio United States; 4 Present Address: Center for Research Excellence in Supportive Care, The Ohio State University James Comprehensive Cancer Center, Columbus, Ohio United States; 5 Present Address: New York State Office for People with Developmental Disabilities, Albany, New York United States; 6 Eshelman School of Pharmacy, University of North Carolina at Chapel Hill, Chapel Hill, USA; 7 UNC Lineberger Comprehensive Cancer Center, Chapel Hill, USA; 8 School of Public Health, University of Pittsburgh, Pittsburgh, USA; 9 School of Social Work, University of Pittsburgh, Pittsburgh, USA; 10 School of Medicine, University of Pittsburgh, Pittsburgh, USA; 11 Present Address: Duke Surgery, Durham, North Carolina United States; 12 Present Address: ImYoo, Mountain View, California United States; 13 Centre for Patient Reported Outcomes Research, University of Birmingham, Birmingham, UK

**Keywords:** Psychometrics, Patient-reported outcome measures, Medication adherence, Oral drug administration, Oral anticancer medications, Multiple myeloma, Cancer

## Abstract

**Background:**

Oral anticancer medications are standard care for cancer. Medication adherence influences health outcomes, but valid, reliable measures assessing self-reported medication adherence are limited. Psychometric properties of the PROMIS^®^ Medication Adherence Scale (PMAS) were evaluated in patients prescribed oral anticancer medications for multiple myeloma.

**Methodology:**

This was a secondary analysis from a longitudinal observational study examining medication adherence, symptoms, quality of life, and financial hardship among individuals prescribed oral anticancer medications for multiple myeloma. PMAS measured self-reported medication adherence. Self-report and medical record data assessed participant characteristics and adherence correlates. Objective medication adherence indices were generated from continuous electronic event monitored data. Internal consistency reliability was estimated using Cronbach’s alpha. Dimensionality was assessed with confirmatory factor analysis. Construct validity was assessed considering adherence correlates. Spearman rank-order correlations summarized associations between PMAS and electronic event monitored data.

**Results:**

PMAS items had limited variability, with high adherence over time. Reliability of PMAS scores was adequate (T1 α = 0.82, 95% CI: 0.75, 0.87; T2 α = 0.84, 95% CI: 0.78, 0.89). Confirmatory factor analysis fit was better for sub-scales (Medication Beliefs and Knowledge and Medication Taking Behaviors) than Total scale, particularly for the Medication Beliefs and Knowledge subscale. Adherence correlates were observed as expected between PMAS and age, self-reported cognitive function, symptom severity, and depression. Weak/moderate positive associations were found between PMAS and electronic event monitored data.

**Conclusions:**

Reliability, two-factor dimensionality, and construct validity were supported, with some evidence of concurrent criterion validity with electronic event monitored adherence data. Additional validation testing is needed to support findings. Evidence supports the feasibility of longitudinal oral anticancer medication adherence assessment monitoring using PMAS.

**Supplementary Information:**

The online version contains supplementary material available at https://doi.org/10.1186/s41687-026-01204-z.

## Background

Oral anticancer medications (OAM) are becoming standard of care for cancer. Between 2000 and 2022, the percentage of oral, versus intravenous, anticancer therapy approvals increased from 7% to 56% [1]. Of new cancer treatments being developed, 40–50% are administered orally [2, 3]. Patients increasingly self-administer OAMs at home, in contrast to intravenous therapies administered by clinicians in clinical settings [3], making medication adherence a critical factor for cancer outcomes in this new treatment paradigm.

Medication adherence across chronic diseases ranges from 46 to 100% and varies widely based on measurement and conceptual approaches [4–7]. Non-adherence (i.e., intentionally or unintentionally not taking one’s medication as prescribed, including delaying, not starting, discontinuing, and/or over/under dosing) [5], has been recognized as a significant clinical problem for some time in chronic disease management. In chronic diseases, poor adherence has been associated with negative effects, including reduced drug effectiveness, quality of life (QoL), and work productivity and including increased symptom burden, morbidity, mortality, health care utilization and costs, and patient and clinician frustration [8–11]. Many types of cancer are now considered chronic diseases and are increasingly being treated with costly, long-term OAMs. As such, non-adherence is gaining renewed and increased attention among cancer researchers and clinicians in this changing paradigm of cancer treatment.

Multiple myeloma (MM) is an incurable chronic blood cancer that requires long-term OAM adherence for optimal outcomes. MM treatment advances, including costly, life-long OAMs, have dramatically improved progression-free and overall survival [12, 13], making OAM adherence critical. Clinical trial findings suggest that taking > 90–95% of doses as prescribed is a predictor of response to treatment in patients with other hematologic malignancies [14, 15].

Patient-reported adherence measures can be helpful when conducting clinical research and assessing patients in practice. A unique advantage of self-report is the ability to reveal non-adherence reasons, prompting timely targeted counseling and intervention. However, there is a critical gap in standardized, clinically feasible, psychometrically valid, and open-access patient-reported adherence measures [16]. The 9-item, Likert scale, Patient-Reported Outcomes Measurement Information System (PROMIS^®^) Medication Adherence Scale (PMAS) was developed and translated into Spanish, Italian, German, and Turkish using rigorous PROMIS methodology to address this gap [17, 18]. Limited studies have conducted psychometric testing on the PMAS after its initial development, including a sample of pregnant patients taking aspirin (*n* = 40) [19] and a sample of kidney transplant recipient taking tacrolimus (*n* = 58; conference abstract) [20]. Additional studies have reported administration of the PMAS to explore medication adherence (e.g., *n* = 11 acute lymphoblastic leukemia pediatric patients taking and *n* = 41 parents administering 6-Mercaptopurine [21]; *n* = 94 chronic myeloid leukemia patients on on kinase inhibitors [TKIs] prescribed antihypertensives [22]; *n* = 58 and *n* = 3 patients prescribed vascular endothelial growth factor [VEGFR] TKIs for renal cell or thyroid cancer, respectively [23]; and *n* = 70 patients prescribed overactive bladder medication [24]). However, the PMAS, to date, has not been psychometrically validated among patients prescribed OAMs for multiple myeloma, a population for whom medication adherence is a vital component of cancer treatment. Therefore, the purpose of this study was to evaluate PMAS psychometric properties through reliability, dimensionality, construct validity, and criterion validity tests using real world data from patients prescribed OAMs as maintenance therapy for multiple myeloma.

## Methods

### Design, setting, sample, and procedures

This was a secondary analysis from an Institutional Review Board approved 6-month observational study (K23NR019296, PI: Belcher) of medication adherence, symptoms, QoL, and financial hardship among patients taking OAMs for MM. Eligibility criteria were age greater than 18 years; prescribed OAM as maintenance therapy for MM; completion of questionnaires in English; and competency to consent. Participants were excluded from final criterion validity analysis if they were not on first- or second-line OAM therapy (lenalidomide or pomalidomide) at enrollment or were missing electronic event monitored (EEM) adherence data.

**Fig. 1:**
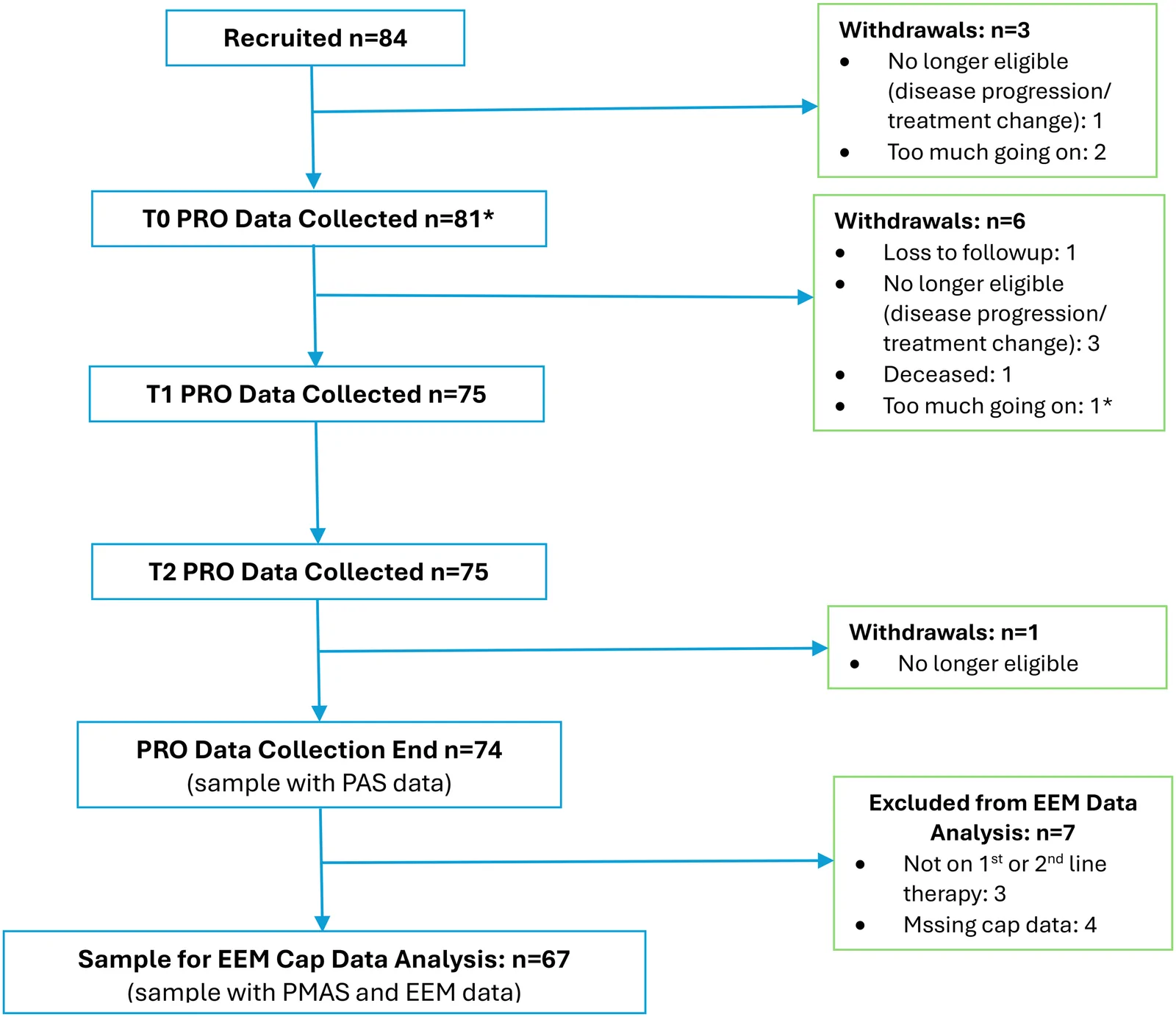
Study schema, illustrating participant recruitment and data collection. Notes. Study time points: T0 = enrollment; T1 and T2 = 3- and 6-month time points following study enrollment, respectively. Abbreviations: PRO = patient reported outcome; EEM =electronic event monitored. One participant voluntarily withdrew after T0 data collection and requested their data be deleted, dropping the T0 sample size to 80

Study enrollment and data collection schema are presented in Fig. 1. Recruitment occurred from four clinics in a Comprehensive Cancer Center in southwestern Pennsylvania. Of the 125 patients approached, 11 (8.8%) were ineligible; 28 (24.6%) of eligible patients declined participation. Reasons for declining were perceived study burden (*n* = 14, 50.0%) and “too much going on” (*n* = 5, 17.9%). Eighty-four patients (73.6% of the 114 approached who were eligible) consented to study participation. Three recruited participants were withdrawn prior to baseline assessment due to treatment plan changes resulting in ineligibility (*n* = 1) or withdrawal (*n* = 2). During the study, 6 (7.1%) participants were withdrawn due to changes in eligibility status, participant request, loss to follow-up, and death. Seventy-four participants (88.1%) had T1 and T2 survey data on the PMAS adherence variables of interest and were included in psychometric testing. A smaller subsample (*n* = 67) had EEM adherence data available and were included in the construct/criterion validity analyses.

Patient-reported and medical record data were collected at enrollment (T0) and at 3- (T1) and 6- (T2) months post study enrollment, 04/2021-03/2023. To increase inclusion and retention [25], different data collection modalities were available, including use of study-provided tablets or participants’ personal devices via electronic links, interview style completion with study staff, or mailed paper packets. Patients were compensated with a $25 gift card for each timepoint completed.

### Measures

*Self-reported medication adherence* was assessed using the PMAS v1.0, a brief, open-access, 9-item Likert scale developed using PROMIS^®^ development procedures [17]. Spaces are provided for the researcher to specify the medication or condition being evaluated and for patients to report the frequency of the prescribed therapy. Each item has 5 response options that vary slightly by subscale, with higher scores indicating better adherence. Subscales scores are generated with a prorated sum of item responses for two scales: Medication Beliefs and Knowledge (MBK; response options: strongly disagree, disagree, neither agree or disagree, agree, or strongly agree; range: 4–20) and Medication Taking Behaviors (MTB; response options: never, rarely, sometimes, almost always, always; range: 5–25), the latter including a 7-day recall period. Subscale scores are summed to generate a Total PMAS score (range: 9–45). The single item, “I took this medication as recommended,” (response options: never, rarely, sometimes, almost always, always) also with a 7-day recall period, is suggested as a measure of Global adherence. The PMAS was administered at the 3- and 6-month follow-up assessments, as some participants had not initiated therapy at study enrollment.

*Sociodemographic and Socioeconomic characteristics* (i.e., age, race, ethnicity, sex, education, annual household income, and a single-item, “Does income meet your basic needs?”) were assessed via self-report [26]. Neighborhood deprivation was assessed using the University of Wisconsin-Madison Area Deprivation Index (ADI) [27, 28], a composite measure of neighborhood socioeconomic status, which includes theoretical domains of income, education, employment, and housing quality. ADI national percentage scores range from 1-100, with higher numbers indicating greater deprivation.

*Clinical characteristics* (i.e., time since diagnosis in months, OAM type by medication name, and time on OAM at enrollment in months) were extracted from medical records using standardized forms.

*Performance status* was assessed using the single item, self-report version of the Eastern Cooperative Oncology Group (ECOG) [29], which assesses level of functioning. Scores range from 0 (no restrictions) to 4 (completely disabled and totally bed/chair confined).

*Symptom severity* was assessed using the revised Edmonton Symptom Assessment Scale (ESAS) [30]. Participants rate their average symptom intensity over 24 h on a 0–10 Likert scale (no symptom to worst possible) for 10 common symptoms experienced by patients with cancer. Items are summed to generate a symptom severity score (0-100).

*Depression*, a common symptom among MM patients [31, 32], as well as a correlate of medication non-adherence [33], was assessed using the Patient Health Questionnaire (PHQ-9) [34]. Respondents report the number of days in the past 2 weeks they experienced depressive symptoms on a Likert scale (0 = not at all to 5 = nearly every day), yielding a 0–27 total sum score. Protocol was in place for participants whose depressive symptom scores reached criteria for moderate depression (PHQ-9 score of 10 or higher). This included notifying the PI of the study within 24 h of completed PHQ-9 assessment, offering mental health resources and referral information, contacting the participant’s physician regarding their score, and documenting any relevant interactions with the participant. Any indication of suicidal ideation prompted immediate contact with the participant, where team members followed an IRB-protocol for differing scenarios based on suicidal ideation severity.

*Cognitive function* was assessed using the 2-item, self-report domain from the European Organization for Research and Treatment of Cancer core questionnaire (EORTC QLQ-C30), version 3.0 [35, 36], which assesses concentration and memory. Items are rated 1 (not at all) to 4 (very much), and scoring procedures transformed data to a 0-100 scale (higher numbers indicating higher/healthier functioning) [37].

*Comorbidity burden* was assessed via the self-reported Brief Charlson Comorbidity Index (CCI) [38, 39]. Participants are provided with a list of 10 comorbidity categories. Endorsed items are weighted to generate a 0–25 scale, where higher scores suggest worse comorbidity burden.

Continuous, *objective medication adherence* data were collected over six months using AARDEX Medication Event Monitoring System (MEMS^®^). MEMS caps contain a microprocessor to capture EEM data, recorded at the time of bottle opening and closing, considered a presumptive dose taken. Participants also completed paper diaries to record the date that the medication was placed in the bottle, when study monitoring began, and to account for known deviations in their MEMS usage (e.g., physician-prescribed drug holiday; did not use while traveling; delayed dose due to forgetting; opening for reasons other than administration). Patient diaries, medical record data, and research field notes reconciled gaps in EEM usage history data and enhanced fidelity. Two objective indices of adherence were calculated from EEM data: (1) percentage of prescribed doses taken and (2) percentage of days with correct intake. We calculated these indices for the first 90-day measurement period, corresponding to the 3-month assessment; the second 90-day period, corresponding to the 6-month assessment; over the full 180-day period; and for 7 days prior to each patient’s PMAS completion, to correspond with the PMAS 7-day recall. EEM data were used as the “gold standard” adherence measure in criterion validity evaluation.

### Data management and statistical analyses

Study methodology and results are reported following Strengthening and Reporting of Observational Studies in Epidemiology (STROBE) guidance [40]. Questionnaire data were stored and managed in REDCap [41, 42]. Scales were scored per developer guidelines. IBM SPSS Statistics, version 29, was used to generate descriptive statistics, correlations, and to test for differences in subsamples. R, version 4.1 psych package, was used for reliability statistics. Mplus, version 8.1 (Muthéen & Muthéen, Los Angeles, CA), was used to assess dimensionality. Statistical significance was set at *p*<.05, two-tailed. SAS, version 9.4 (SAS Institute, Inc., Cary NC), was used for EEM data management and analyses.

### Descriptive statistics

Descriptive statistics for total sample and the subsample with EEM data were calculated, using frequency/percentage, mean/standard deviation/range, or median/interquartile range/range as appropriate for each variable.

### Psychometric evaluation

#### Reliability

We computed Cronbach’s alpha, with 95% confidence intervals, to estimate reliability of the unweighted PMAS sum scores. We utilized the conventional criteria of α ≥ 0.7 being considered adequate, α ≥ 0.8 as good, and α ≥ 0.9 as excellent [43]. McDonald’s Omega estimated the reliability of weighted scale scores. Spearman rank-order coefficient was computed to assess consistency in the scale and sub-scale scores between time points, as the score distributions exhibited notable skewness. Item fit is considered in relation to the full and sub-scale by computing item-test correlations along with change in alpha if item is dropped.

#### Dimensionality

To assess dimensionality, we conducted confirmatory factor analyses (CFA) at each time point for the unidimensional model of Total PMAS factor, and the hypothesized MTB and MBK subscale factors. We present model fit indices Comparative Fit Index (CFI) and Root Mean Squared Error of Approximation (RMSEA) with 90% confidence intervals and Bayesian Information Criteria (BIC) to determine how well these specific factor structures fit the data [44, 45]. Lower values of BIC and RMSEA and higher values of CFI indicate better fit of given factors to item response data. Full scale factor showing better fit than the sub-scale factors suggest it may be more valid to report full scale over sub-scales. Where individual sub-scales exhibit poor fit, it suggests that sub-scale scores may not be valid. We do at each time point to see if there is drift in fit over time. We employed Bayesian estimation with empirically informed priors (See Table S1 for model specification details), as this is known to perform better than maximum likelihood with smaller sample sizes [46, 47]. We report CFI and RMSEA with 90% confidence intervals for the selected models to provide relative and absolute fit indices. Hu & Bentler’s criteria [48], CFI ≥ 0.95 and RMSEA close to 0.06 or less, concluded whether the hypothesized factor structure provided a close fit to the observed data.

#### Construct validation

To examine convergent construct validity evidence, we tested whether factors associated with adherence [17, 33] demonstrated expected relationships. Due to PMAS score skew and outliers, we used Spearman’s rank-order correlation. The known factors associated with adherence that were available in the dataset, derived from self-report, that we considered were age, sex, race, income, and education; cognitive function; symptom severity; depression; physical function; and comorbidities.

#### EEM criterion validity

To assess concordance of PMAS scores with an objective adherence measure, we evaluated Spearman rank-order correlations (rho) to summarize associations between PMAS scores at 3- and 6-month study time points of administration and EEM doses and days adherence indices, based on the first 90 days, second 90 days, total 180 days of study monitoring, and the seven day period prior to PMAS survey completion, to correspond with PMAS recall period evaluated for the MTB subscale and Global adherence single-item.

## Results

### Participants

Participant sociodemographic and clinical characteristics and scale scores are reported for the total sample of participants and the subsample with both PMAS and EEM adherence data (Table 1). The total sample was on average 63.8 years old, included more men than women (59.5%), and mostly identified as either non-Hispanic white (83.3%) or Black/African American (13.5%). A range of educational attainment, income, and neighborhood deprivation values were represented. Most participants (97.2%) reported income meeting their basic needs. Average ECOG performance status was 1. Most participants were 33.5 months since MM diagnosis (IQR = 50.75, range: 6-226 months), prescribed front- or second-line OAM (66.2% lenalidomide, 29.7% pomalidomide) for a median treatment time of 11.0 months (IQR = 22, range: 0–78 months). Average self-reported scale scores were symptom severity = 16.4; depression = 3.4 (no participants at any timepoint reported positive suicidal ideation); cognitive function = 84.7; and comorbidity burden = 5.4. For the subsample whose data were used in PMAS and EEM correlational analyses, characteristics were similar, with a slightly higher percentage of patients prescribed lenalidomide.

**Table 1:** Participant characteristics and scale scores for the total sample of participants with PMAS data versus the subsample of participants with PMAS and EEM data

Characteristic	Total Sample (*n* = 74) Mean ± SD, Range or *n* (%)	Subsample with EEM Data (*n* = 67) Mean ± SD, Range or *n* (%)
Age, years	63.8 ± 10.3, 35–90	63.9 ± 10.6, 35–90
Ethnicity and Race		
Non-Hispanic, White	62 (83.3)	58 (86.6)
Non-Hispanic Black or African American	10 (13.5)	7 (10.4)
Hispanic, “other” race	2 (2.7)	2 (3.0)
Sex Assigned at Birth/Gender		
Male	44 (59.5)	38 (56.7)
Female	30 (40.5)	29 (43.3)
Education, highest level completed		
≤ High School Graduate or Equivalent	15 (20.3)	14 (20.9)
Some College / Vocational-Technical Training / Associate Degree	24 (32.4)	22 (32.8)
Bachelor’s Degree or more	35 (47.3)	31 (46.3)
Income, annual household (*n* = 55)		
< $50,000	15 (27.3)	13 (26.5)
$50,000 - <$90,000	14 (25.5)	14 (28.6)
$90,000 - $150,000	19 (34.5)	18 (36.7)
> $150,000	7 (12.7)	4 (8.2)
Income meets basic needs		
Yes	69 (97.2)	63 (98.4)
No	2 (2.8)	1 (1.6)
Neighborhood Deprivation, national percentile (ADI)	58.7 ± 23.0, 7-100	58.0 ± 23.0, 7-100
Performance Status (ECOG)	0.89 ± 0.69, 0–3	0.91 ± 0.71, 0–3
Time Since Diagnosis, months (median [IQR], range)	33.5 (50.75), 6-226	35 (57), 6-226
OAM Therapy at Enrollment		
Lenalidomide	49 (66.2)	47 (70.1)
Pomalidomide	22 (29.7)	20 (29.9)
Ixazomib	3 (4.1)	0 (0.0)
Time on OAM at Enrollment, months (median [IQR], range)	11.0 (22), 0–78	11.0 (22), 0–78
Symptom Severity (ESAS sum score)	16.4 ± 13.5, 0–53	16.2 ± 13.4, 0–53
Depression (PHQ-9)	3.4 ± 3.2, 0–13	3.4 ± 3.2, 0–13
Cognitive Function (EORTC subscale)	84.7 ± 17.2, 16.7–100	85.1 ± 17.0, 16.7–100
Comorbidity Burden (CCI)	5.4 ± 9, 0–11	5.4 ± 2.8, 0–11

Notes. PMAS versus PMAS and EEM columns represents summary statistics for the subsamples of participants who had self-reported PMAS adherence data versus both PMAS and EEM data, respectively. Abbreviations: PMAS = PROMIS Medication Adherence Scale; EEM = electronic event monitoring; SD = standard deviation; ADI = Area Deprivation Index; ECOG = Eastern Cooperative Oncology Group; OAM = oral anticancer medication; IQR = inter-quartile range; EORTC = European Organization for Research and Treatment of Cancer; ESAS = Edmonton Symptom Assessment Scale; PHQ = Patient Health Questionnaire; CCI = Charlson Comorbidity Index

Descriptive statistics for PMAS item responses at both the 3- and 6-month time points, T1 and T2 along with a simple difference score in the total PMAS scores calculated on the complete cases as T2-T1, are presented as additional material (Table S1). Little variation existed amongst item responses, with most participants reporting high adherence. Aside from item 4, “I believe this medicine is working,” at T2, at least 75% of the respondents rated items at “5,” indicating most adherent. This was reinforced by the scores on the Global item for adherence and total and subscale scores (Table S2). On the Global item, which asked respondents to rate, “In the past 7 days… I took this medicine as prescribed” on a never (1) to always (5) Likert scale, over 80% of respondents responded “5” at both time points.

Most participants exhibited little to no change in total PMAS scores over time (Tables 2 and 3), with 44% exhibiting no change, 31% having a decrease, and 25% showing an increase. There were extreme cases, with four people having more than a 5-point decline in PMAS total score and three people having more than a 5-point increase in PMAS total score. Amongst those showing a decrease, the average score went from 44.14 (SD = 1.49) at T1 to 40.23 (SD = 5.14) at T2. Amongst those showing an increase, the average went from 40.22 (SD = 5.52) at T1 to 44.33 (SD=0.77) at T2. Scores clustered at the upper end of adherence at both time points, indicating ceiling effect presence.

**Table 2:** PROMIS Medication Adherence Scale change scores, at 3- and 6-months after participant study enrollment

Sub-scales	Item	Mean	SD	Min	25p	Median	75p	Max	Mode
**T1: 3-month (*N*** **= 72)**									
MBK	1	4.78	0.83	1	5	5	5	5	5
	2	4.72	0.84	1	5	5	5	5	5
	3	4.75	0.85	1	5	5	5	5	5
	4	4.64	0.91	1	5	5	5	5	5
MTB	5	4.81	0.43	3	5	5	5	5	5
	6	4.78	0.45	3	5	5	5	5	5
	7 (rev)	4.90	0.34	3	5	5	5	5	5
	8 (rev)	4.99	0.12	4	5	5	5	5	5
	9 (rev)	4.92	0.52	1	5	5	5	5	5
Overall Global Rating		4.81	0.43	3	5	5	5	5	5
**T2: 6-month (*N*** **= 74)**									
MBK	1	4.80	0.81	1	5	5	5	5	5
	2	4.78	0.73	1	5	5	5	5	5
	3	4.76	0.76	1	5	5	5	5	5
	4	4.54	0.83	1	4	5	5	5	5
MTB	5	4.78	0.50	2	5	5	5	5	5
	6	4.84	0.37	4	5	5	5	5	5
	7 (rev)	4.91	0.34	3	5	5	5	5	5
	8 (rev)*	4.96	0.26	3	5	5	5	5	5
	9 (rev)*	4.95	0.37	2	5	5	5	5	5
Overall Global Rating		4.78	0.50	2	5	5	5	5	5

Note. *Sample size for items 8 and 9 at T2 is 73, as one patient had missing data for these items. Abbreviations: SD = Standard Deviation; rev = reverse (scored); MBK = Medication Beliefs and Knowledge subscale; MTB = Medication Taking Behaviors subscale

**Table 3:** PMAS scores at 3- and 6-months after enrollment, with changes in Total Score over time

	Mean	SD	Min	25p	Median	75p	Max
T1							
Total	43.30	3.67	28	43	45	45	45
MBK	18.90	3.30	4	20	20	20	20
MTB	24.40	1.22	18	24	25	25	25
T2							
Total	43.15	3.72	27	43	45	45	45
MBK	18.88	2.964	4	19	20	20	20
MTB	24.27	1.86	13	24	25	25	25
Change in Total from T1 to T2							
T2-T1	-0.17	4.72	-17	-1	0	0.5	16

Note. Abbreviations: PMAS = PROMIS Medication Adherence Scale; SD = Standard Deviation; MBK = Medication Beliefs and Knowledge subscale; MTB = Medication Taking Behaviors subscale

### Psychometric

#### Reliability

Items associated with the MBK subscale exhibited strong correlations among responses, indicating high internal consistency, while the MTB items exhibited smaller correlations, suggesting lower internal consistency (see additional Table S3 for PMAS item response correlations by time point). The reverse coded items had weaker correlation to other responses but showed more moderate correlations with one another.

Internal consistency findings of PMAS items by time point are presented in Table 4. Item-test correlations and change to internal consistency if an item is dropped are presented for subscales (Table 5) to reflect consistency in responses to each item with other items and reflect how well each item contributes to the overall scale. In general, items associated with the MBK subscale tend to fit better than those associated with MTB, suggesting higher measurement error due to this set of subscale items. Supplemental Figure S1 gives additional information demonstrating how well items load onto their respective scales. The MBK and MTB confidence interval overlaps across time points, suggesting reliability for each subscale was consistent over time. All of the confidence intervals contained a value of α = 0.7; by Nunnally’s criteria, this is considered adequate for early stages of research [43].

**Table 4:** Internal consistency of PROMIS Medication Adherence Scale items by time point

Item	T1 (3 month): α = 0.82, 95% CI: 0.74, 0.87		T2 (6 month): α = 0.84, 95% CI: 0.78, 0.89	
	Item-Test Correlation	Alpha if item is dropped	Item-Test Correlation	Alpha if item dropped
1	0.83	0.75	0.76	0.80
2	0.86	0.74	0.81	0.79
3	0.90	0.74	0.86	0.78
4	0.86	0.74	0.80	0.79
5	0.37	0.81	0.36	0.84
6	0.10	0.83	0.33	0.84
7	0.11	0.83	0.23	0.85
8	0.34	0.82	0.37	0.84
9	0.12	0.84	0.34	0.84

**Table 5:** Internal consistency of PROMIS Medication Adherence Scale subscale items

Item	Medication Beliefs and Knowledge (MBK) subscale			
	T1 (3 month): α = 0.98, 95% CI: 0.97, 0.99		T2 (6 month): α = 0.96, 95% CI: 0.94, 0.97	
	Item-Test Correlation	Alpha if item is dropped	Item-Test Correlation	Alpha if item dropped
1	0.94	0.97	0.86	0.95
2	0.95	0.97	0.92	0.93
3	0.97	0.96	0.95	0.92
4	0.92	0.98	0.84	0.96

	Medication Taking Behavior (MTB) subscale			
	T1 (3 month): α = 0.59, 95% CI: 0.42, 0.72		T2 (6 month): α = 0.73, 95% CI:0.62, 0.82	
5	0.52	0.43	0.71	0.59
6	0.34	0.54	0.26	0.77
7	0.32	0.55	0.26	0.76
8	0.56	0.56	0.75	0.63
9	0.30	0.58	0.65	0.62

#### Dimensionality

**Fig. 2:**
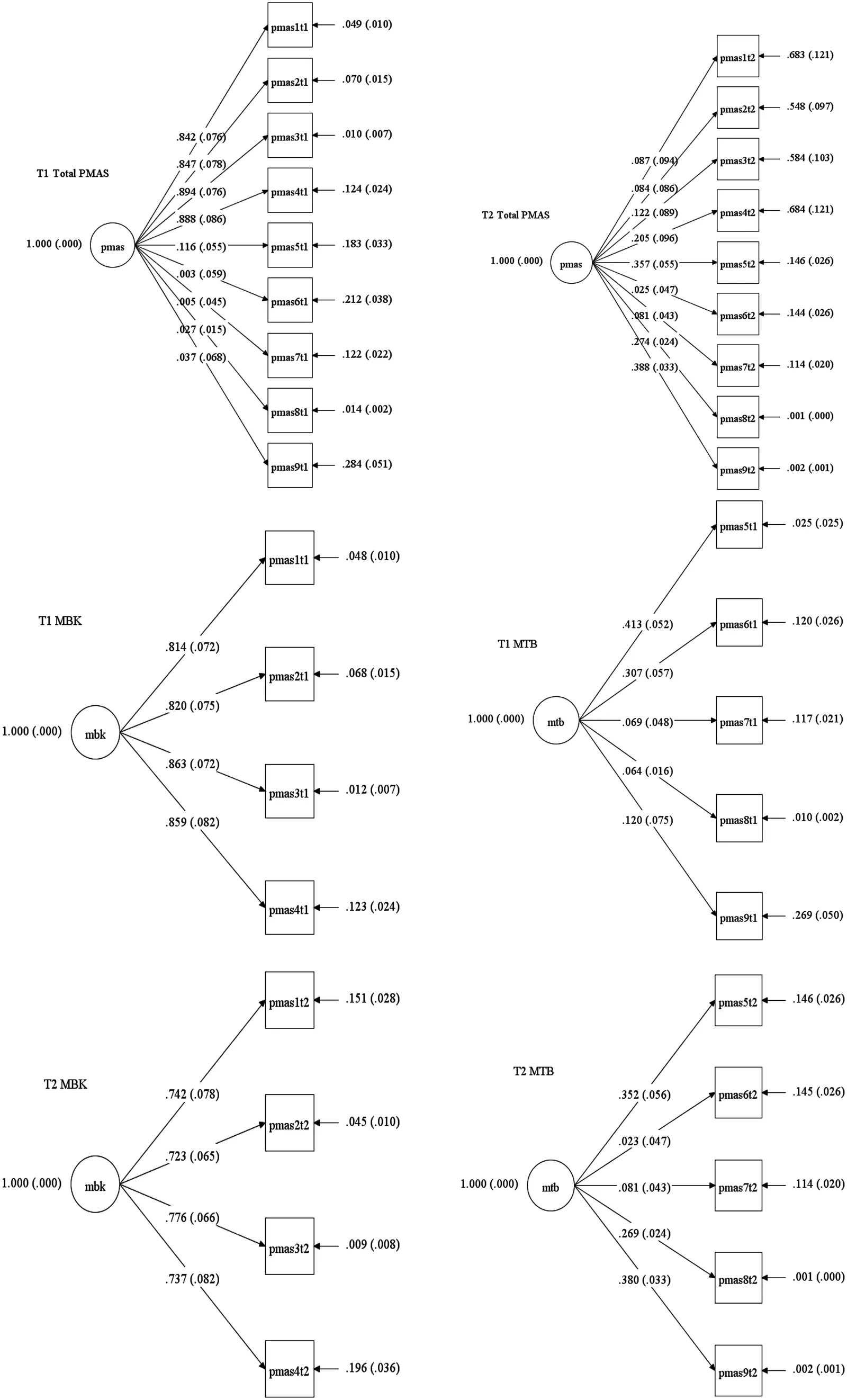
Factor loadings for each PMAS item [Total, MTB, MBK] at the T1and T2 time points. Notes. T1 (3-month) on left, T2 (6-month) on right

BIC was notably higher (i.e., worse fit) for the one factor total score model than the subscales at both time points. For the most part fit indices are poor, however, the MBK fits indicate this scale performing well, especially at time 2, however there is some concern at time 2 that we may have an overfit solution given that CFI = 1 and RMSEA = 0, despite BIC being higher than it was T1 for this subscale. The MTB scale appears to be very weak, and the full scale does not reach conventional criteria for good fit either. Providing validity evidence for the MBK sub-scale and issues with the MTB subscale. The degraded fit for the full scale is likely attributable to the very poor fit for the MTB subscale. Table 6 displays model fit indices for each subscale and total score models. According to common criteria [48] for good model fit, only the MBK subscale at time 2 fully meets good fit; while at time 1, fit appears good, in line with CFI, it does not with RMSEA. See Fig. 2 for factor loadings for each item on the scale at each time point, along with their posterior standard deviations, reflecting the degree to which responses to specific items are associated with MTB and MBK subscales.

**Table 6:** Model fit indices for the total PMAS and MBK and MTB subscales over time

Model	CFI	90% CI		RMSEA	90% CI		BIC
		LCL	UCL		LCL	UCL	
T1 PMAS	0.802	0.777	0.821	0.24	0.229	0.255	578.791
T2 PMAS	0.412	0.393	0.427	0.465	0.46	0.72	754.802
T1 MBK	0.975	0.954	0.988	0.245	0.17	0.332	296.973
T2 MBK	1	0.973	1	0	0	0.214	362.733
T1 MTB	0.686	0.543	0.784	0.246	0.206	0.296	226.863
T2 MTB	0.832	0.804	0.851	0.372	0.35	0.401	21.225

Note. Abbreviations: PMAS = PROMIS Medication Adherence Scale; MBK = Medication Beliefs and Knowledge subscale; MTB = Medication Taking Behaviors subscale; CI=Confidence Interval; LCL=Lower Confidence Level; UCL=Upper Confidence Level; CFI=Comparative Fit Index; RMSEA= Root Mean Squared Error of Approximation; BIC= Bayesian Information Criteria

#### Known factors associated with adherence

To validate that scores from PMAS reflect expected relationships between adherence and factors described as correlates in the literature [17, 33], we report significance tests and effect sizes in Table 7. The statistically significant associations identified were in the hypothesized directions: (1) We observed positive associations between higher PMAS scores and both higher age and better self-reported cognitive function. (2) We observed negative associations between lower PMAS scores and both increased symptom severity and higher depression. Sample data did not provide strong evidence for associations between PMAS and sex, race, income, education, physical function, or self-reported comorbidities.

**Table 7:** Associations between PMAS scores and factors from literature known to be associated with adherence

Variable	Measure	T1 (3 month)	T2 (6 month)
Age (years)	Self-report	MBK: rho=0.122 MTB: rho=0.124 Total: rho=0.154	MBK: rho=0.227* MTB: rho=0.270* Total: rho=0.300*
Sex (women vs. men)	Self-report	MBK: rho=0.100 MTB: rho=-0.171 Total: rho=-0.064	MBK: rho=0.077 MTB: rho=-0.060 Total: rho=-0.050
Race (white vs. other)	Self-report	MBK: rho=0.098 MTB: rho=0.068 Total: rho=0.083	MBK: rho=0.096 MTB: rho=0.055 Total: rho=0.172
Income	Self-report	MBK: rho=-0.015 MTB: rho=-0.085 Total: rho=-0.106	MBK: rho=-0.107 MTB: rho=0.075 Total: rho=-0.054
Education	Self-report	MBK: rho=-0.153 MTB: rho=-0.167 Total: rho=-0.190	MBK: rho=0.159 MTB: rho=0.036 Total: rho=0.183
Comorbidities	CCI	MBK: rho=-0.197* MTB: rho=-0.057 Total: rho=-0.108	MBK: rho=0.014 MTB: rho=-0.137 Total: rho=-0.088
Physical function	EORTC, domain score	MBK: rho=0.170 MTB: rho=0.137 Total: rho=0.191	MBK: rho=0.027 MTB: rho=-0.038 Total: rho=0.030
Cognitive function	EORTC, domain score	MBK: rho=0.260* MTB: rho=0.268* Total: rho=0.308*	MBK: rho=0.224* MTB: rho=0.229* Total: rho=0.294*
Symptom severity	ESAS, sum score	MBK: rho=-0.273* MTB: rho=-0.171 Total: rho=-0.291*	MBK: rho=-0.268* MTB: rho=-0.113 Total: rho=-0.239*
Depression	PHQ-9, score	MBK: rho=-0.355* MTB: rho=-0.154 Total: rho=-0.257*	MBK: rho=-0.307* MTB: rho=-0.128 Total: rho=-0.291*

Note. *indicates statistically significant relationships with *p* < .05 (one-tailed in direction of expected relationships). Abbreviations: PMAS = PROMIS Medication Adherence Scale; MBK = Medication Beliefs and Knowledge subscale; MTB = Medication Taking Behaviors subscale; CCI = Charleson Comorbidity Index, EORTC = European Organization for Research and Treatment of Cancer; ESAS = Edmonton Symptom Assessment Scale; PHQ = Patient Health Questionnaire

#### EEM concordance testing

Table 8 reports Spearman rank-order correlations between PMAS scores and adherence indices generated from EEM data during the first 90 days, second 90 days, the entire monitoring period, and the 7 days prior to each participant’s PMAS assessment, to correspond with the recall period included with the items for the MTB subscale and Global adherence item. The median (IQR) adherence based on EEM over the period of follow-up was 88.1 (IQR = 27.51) for the percentage of prescribed doses taken (“doses EEM adherence”) and 86.6 (IQR = 25.96) for the percentage of day with correct intake (“days EEM adherence”). Statistically significant, weak to moderate, positively associated relationships were observed between both doses and days EEM adherence indices and PMAS scores, mainly in the PMAS scores with behavioral items (Total score, MTB subscale, and Global item). Evaluation of the 7-day window of EEM data prior to PMAS adherence assessment did not identify statistically significant relationships between PMAS and EEM data.

**Table 8:** Correlations (Spearman rank-order) between PMAS scores and EEM medication adherence data

Time Point	PMAS Measure	DOSES EEM Adherence				DAYS EEM Adherence			
		1st 90 days	2nd 90 days	180 days	7-days	1st 90 days	2nd 90 days	180 days	7-days
3-month	Total	0.23	0.31*	0.32**	0.06	0.23	0.27*	0.30*	0.10
	MBK	0.03	0.19	0.15	− 0.12	0.06	0.16	0.14	− 0.04
	MTB	0.32*	0.29*	0.34**	0.11	0.29*	0.24	0.32**	0.17
	Global	0.19	0.32*	0.27*	− 0.01	0.17	0.27*	0.25*	0.01
6-month	Total	0.12	0.41**	0.28*	0.01	0.17	0.33**	0.27*	0.05
	MBK	− 0.10	0.26*	0.12	− 0.15	− 0.06	0.21	0.11	− 0.07
	MTB	0.23	0.29*	0.24*	0.24	0.24	0.22	0.22	0.15
	Global	0.10	0.19	0.12	0.18	0.17	0.13	0.13	0.11

Notes. *p<.05; **p<.01

## Discussion

This is the first study to report PMAS psychometric properties using clinical data collected from patients prescribed OAMs among patients with cancer, specifically with MM. There was little change in PMAS scores over time. In the context of a sample that self-reported high OAM adherence and data that suggested ceiling effects, we found evidence supporting reliability at both time points and two-factor dimensionality. We also found evidence confirming relationships between PMAS scores and factors described in the literature as relating to adherence and concordance of PMAS scores with adherence indices based on EEM data.

Variability in PMAS scores was limited among study participants. Item 4, “I believe this medication is working,” was the only item for which 75% of respondents did NOT rate the item at a 5. It is possible that the lower score is a function of this terminal cancer that frequently evades treatment. High scores overall may also suggest that patients know how critical it is to adhere to their OAM therapy, given the high potential consequence of nonadherence in the context of cancer treatment, or desirability bias that is inherent in self-report measures.

It is expected that the correlations between item responses will be high for the MBK subscale items (1–4), and lower for the MTB subscale items (5–9). It is arguable that the items in the MTB scale are more formative indicators, representing an index of behaviors constituting adherence, rather than reflective indicators representing a latent trait. The formative indicator view of MTB does not assume that the different behaviors are correlated with each other, reflecting an underlying construct for belief and knowledge. This is likely why fit indices only look good for MBK but not MTB or total. Of note, for MTB at T1, McDonald’s omega of 0.89 falling above the confidence interval for Cronbach’s alpha suggests that weighted composites may be a more reliable approach to scoring MTB.

The generally accepted definition of adherence is the extent to which patients take their medications as prescribed, with adherence quantification represented by summary statistics [5]. Though both PMAS and EEM are adherence measures, an expectation of very high correlations as a test of validity may not make sense conceptually, as they are assessing different aspects. Excluding the Global adherence item (MedAd5), most PMAS items are not about precise correspondence between the medication taking behavior and their prescription. They are more about beliefs, attitudes, and reasons for not taking medications in general. In addition, the beliefs and attitudes questions have no recall period, while the behaviors questions have a 7-day recall. It is not clear if this would correspond to a 90-day or 180-day trend from EEM, even if it was expected that the two measures would be well-correlated. Our EEM concordance testing, using EEM findings as the “gold standard” measure for comparison to PMAS measure findings did not show evidence of particularly high correlations, suggesting that one measure should not be substituted for another. We do not consider the two measures to be interchangeable and suggest that the two measures may be capturing meaningfully different aspects of OAM adherence. That said, the correlations are far from trivial, and more of a construct validity test (vs. criterion), and could be considered moderate.

Adherence rates for patients taking OAMs for MM vary. A 2022 systematic review and meta-analysis of adherence to and persistence with OAMs in patients with MM found that the pooled proportion of patients who were adherent to therapies was 67.9% (range: 29–98%) [49]. When they compared self-report to medication possession ratio (MPR) data, the proportion of adherent patients was higher, 81.6% versus 61.0%. Reviews that reported factors associated with nonadherence identified increasing age, higher comorbidity, polypharmacy, and poor social support. Another 2023 study of participants prescribed lenalidomide for MM compared adherence data over three months using multiple measurements (i.e., MEMS caps, MPR, and self-report via the Brief Adherence Rating Scale [BARS]) [50]. Authors estimated adherence to lenalidomide among these patients to be 98% using MEMS caps; somewhat lower using MPRs, as MPRs did not account for prescribed medication holds; and 100% using BARS.

Our findings were consistent with available findings in other validation studies that determined PMAS to be valid and reliable. The total mean PMAS score in our study, 44.14 (SD = 1.49), was higher than both the Ruderman study of pregnant patients taking aspirin (41.18 [SD = 3.90]) [19] and the Peipert study of kidney transplant recipients taking tacrolimus (38.7 [SD = 2.2]) [20]. MBK and MTB subscale and single item (MedAd5) means as well as Chronbach alpha for reliability in our study at both time points were similar to the findings in the single time point of the Ruderman study. Additional research is needed to further elucidate the clinical meaning of these values as well as whether a clinical cut point can be identified to signal increased risk for nonadherence. As previously outlined, use of the PMAS to measure adherence also been explored in other populations [21–24]. In the study of acute lymphoblastic leukemia patients taking 6-mercaptopurine (*n* = 11; 1–17 years of age) and their parents (*n* = 41), authors reported that PMAS scores did not correlate with a visual analogue scale of adherence (VAS_dose_), but PMAS scores were not reported [21]. In the study of CML patients on TKI’s prescribed antihypertensives, authors reported that high self-reported adherence scores, as assessed by the Adherence to Refills and Medications Scale (ARMS-7), were corroborated by the PMAS, but scores were again not reported [22]. Psychometric testing of the PMAS across varying populations remains a clinical and research need.

An examination of adherence measures in other conditions indicates similar variability in estimates of adherence between measures [51, 52]. This variation by measurement methods creates considerable complexity in selecting measures and interpreting adherence data. For example, measurement methods may have differing associations with clinical outcomes [53]. Further, different adherence measures tend to be associated with different predictors or correlates of adherence [33], supporting the need for multi-modal assessments in the evaluation of adherence.

### Strengths, limitations, and research implications

A main study strength was the real-world data application to psychometric testing of an open access measure of self-reported adherence. While data were collected during the COVID-19 pandemic, research was permitted to continue with safety precautions, and enrollment goals were met. Findings provide evidence to generate hypotheses and inform adherence measurement for cancer and chronic disease populations.

Authors acknowledge study limitations. The sample size was relatively small and included some missing EEM data; however, robust statistical methods were employed to account for this, and the subsample was relatively like the full sample. While recruitment and data collection were designed to be generalizable, small cell sizes prevented investigation of potential biases introduced by using multiple modes of data collection and variability by recruitment site. Further, administration of the PMAS and other self-reported instruments at the 3- and 6-month study time points limited our ability to detect more granular changes assessed by continuous EEM data. Adherence contextual factors among the cancer population may be unique. While OAMs are costly, people may be more likely to adhere to a potentially life sustaining medication. Further, MM is a rare cancer, limiting the ability to generalize findings. Lastly, our study made use of numerous self-reported variables to describe our sample and study correlates; future studies should balance participant burden with use of a more comprehensive assessment of cognitive function that includes both self-report and objective measures.

Additional testing is needed in larger studies with sociodemographically and clinically diverse samples to support findings. With diverse samples, we can identify measurement biases and adapt instruments for more accurate capture of patient perspectives [54]. Prospective studies with repeated patient-reported adherence outcomes may be a rich source of data to link with objective clinical assessments over the treatment trajectory course, but clinical researchers must balance these potential research benefits with the response burden placed on patients when asking them to complete repeated assessments. Future studies should also examine incidences of false positives and negatives in predicting EEM adherence levels, and future correlations between PMAS and EEM data should incorporate the self-reported recall period into analyses. Qualitative data should also be incorporated into the assessment of this instrument to investigate whether PMAS items represent the content areas that are most clinically relevant and important to patients.

### Clinical and policy implications

As chronic disease incidence increases globally, medication adherence is a public health and clinical priority. With increasing options for OAMs, multi-level, targeted interventions are needed to support medication adherence. The PMAS is an inexpensive, non-invasive tool to assess medication adherence that can be integrated with health records. With additional testing and refinements, the PMAS offers researchers and clinicians an open access, clinically relevant instrument to assess medication adherence. PMAS administration may identify medication adherence barriers, guide intervention development, and improve clinical care to increase adherence and optimize patient outcomes.

Coupling the advancement of methodologies that individualize cancer treatment strategies [55] with the expansion of OAMs in standard treatment regimens, medication adherence plays a large role in treatment efficacy. In hematologic malignancies requiring OAMs, medication adherence is a critical determinant of treatment effectiveness. For example, in a cohort of 1,351 MM patients [56], MPR ≤ 0.8 to lenalidomide treatment was associated with higher likelihood of progression and death. Similar findings have been found with BCR-ABL inhibitors in chronic myeloid leukemia [14, 57], BTK-inhibitors in chronic lymphocytic leukemia [15], and mercaptopurine maintenance in acute lymphoblastic leukemia [58].

Although oncology professional societies have made recommendations about monitoring adherence, there are no widely used adherence measures feasible for clinical contexts [59]. In addition to PMAS, a recent study reported on the development and validation of another self-reported adherence to cancer therapy measure, Domains of Subjective Extent of Nonadherence (DOSE-Nonadherence) [60]. DOSE-Nonadherence is intended for use among heterogeneous cancer populations, treatment types, and administration settings and identifies self-reported nonadherence reasons. Both measures address a critical need to evaluate medication adherence and warrant additional validation and testing prior to use in real-world clinical settings.

### Conclusions

In the context of broader cancer and chronic disease adherence literature, findings support the need for multi-modal adherence assessments. Although self-reported adherence measures are important in routine clinical care, periodic assessment of objective adherence (e.g., refill records and pill-counts) should be considered. Our study demonstrated feasibility of collecting patient-reported OAM adherence via PMAS at two time points, providing evidence that PMAS assessment can be integrated into longitudinal patient monitoring.

## Supplementary Information

Below is the link to the electronic supplementary material.

Supplementary Material 1

## Data Availability

This study and the analysis plan were not formally registered/pre-registered. De-identified data and analytic code used to conduct analyses presented in this study are not available in a public archive but may be made available, as allowable according to institutional IRB standards, by emailing the corresponding author. Some of the materials (i.e., survey instruments) used to conduct the study are publicly available by contacting the instrument developers.

## Abbreviations

OAM: Oral anticancer medication
QoL: Quality of life
MM: Multiple myeloma
PROMIS^®^: Patient–Reported Outcomes Measurement Information System
PMAS: PROMIS^®^ Medication Adherence Scale
EEM: Electronic event monitored
MBK: Medication Beliefs and Knowledge
MTB: Medication Taking Behaviors
ADI: Area Deprivation Index
ECOG: Eastern Cooperative Oncology Group
ESAS: Edmonton Symptom Assessment Scale
PHQ–9: Patient Health Questionnaire
EORTC QLQ–C30: European Organization for Research and Treatment of Cancer core questionnaire
CCI: Charlson Comorbidity Index
MEMS®: Medication Event Monitoring System
STROBE: Strengthening and Reporting of Observational Studies in Epidemiology
CFA: Confirmatory factor analysis
CFI: Comparative fit index
RMSEA: Root mean squared error of approximation
BIC: Bayesian Information Criteria
CI: Confidence interval
LCL: Lower confidence level
UCL: Upper confidence level
MPR: Medication possession ratio
BARS: Brief Adherence Rating Scale
DOSE: Nonadherence–Domains of Subjective Extent of Nonadherence
