# Supplementary material for: Psychometric evaluation of the PROMIS® Medication Adherence Scale among patients prescribed oral anticancer medication for multiple myeloma
Source: J Patient Rep Outcomes. 2026 Sep 19;10:172. doi: 10.1186/s41687-026-01204-z (PMC13615244; doi:10.1186/s41687-026-01204-z)
Supplement: Supplementary file 1 [file 41687_2026_1204_MOESM1_ESM.docx]

**Psychometric Evaluation of the PROMIS® Medication Adherence Scale (PMAS) among Patients on Oral Anticancer Medication for Multiple Myeloma**

**Supplementary Materials**

| Table S1: Details on Specifications for fitting model with Bayesian Estimation | |
| --- | --- |
| MCMC Algorithm | Gibbs Sampler |
| Starting Values | Maximum Likelihood Estimates |
| Chains | 4 |
| Iterations per Chain | 25,000 |
| Burn-in per Chain | 12,500 |
| Thinning | 10 |
| Fixed Factor Parameters | N(0,1) |
| Model Priors: | Informed by Random 20% Sub-Sample |
| Loadings on MBK | N(0.8,0.2) |
| Loadings on MTB | Items 6 & 7: N(0.05,0.25)  Otherwise: N(0.3,0.2) |
| Intercepts | N(5, 0.5) |
| Residuals | IG(4,3) |

| Table S2. Descriptive statistics for the PROMIS Medication Adherence Scale item responses at 3- and 6-month study time points. | | | | | | | | | |
| --- | --- | --- | --- | --- | --- | --- | --- | --- | --- |
| **Time 1: 3 months** | **N=73** | |  |  |  |  |  |  |  |
| **Subscales** | **Item** | **Mean** | **SD** | **Min** | **25p** | **Median** | **75p** | **Max** | **Mode** |
| MBK | 1 | 4.78 | 0.82 | 1 | 5 | 5 | 5 | 5 | 5 |
|  | 2 | 4.73 | 0.84 | 1 | 5 | 5 | 5 | 5 | 5 |
|  | 3 | 4.75 | 0.85 | 1 | 5 | 5 | 5 | 5 | 5 |
|  | 4 | 4.64 | 0.90 | 1 | 5 | 5 | 5 | 5 | 5 |
|  | 5 | 4.81 | 0.43 | 3 | 5 | 5 | 5 | 5 | 5 |
| MTB | 6 | 4.78 | 0.45 | 3 | 5 | 5 | 5 | 5 | 5 |
|  | 7 (rev) | 4.90 | 0.34 | 3 | 5 | 5 | 5 | 5 | 5 |
|  | 8 (rev) | 4.99 | 0.12 | 4 | 5 | 5 | 5 | 5 | 5 |
|  | 9 (rev) | 4.92 | 0.52 | 1 | 5 | 5 | 5 | 5 | 5 |
| Overall Global Rating | 4.81 | | 0.43 | 3 | 5 | 5 | 5 | 5 | 5 |
| **Time 2: 6 months** | **N=73** | |  |  |  |  |  |  |  |
| **Subscales** | **Item** | **Mean** | **SD** | **Min** | **25p** | **Median** | **75p** | **Max** | **Mode** |
| MBK | 1 | 4.80 | 0.81 | 1 | 5 | 5 | 5 | 5 | 5 |
|  | 2 | 4.78 | 0.73 | 1 | 5 | 5 | 5 | 5 | 5 |
|  | 3 | 4.76 | 0.76 | 1 | 5 | 5 | 5 | 5 | 5 |
|  | 4 | 4.54 | 0.83 | 1 | 4 | 5 | 5 | 5 | 5 |
| MTB | 5 | 4.78 | 0.50 | 2 | 5 | 5 | 5 | 5 | 5 |
|  | 6 | 4.84 | 0.37 | 4 | 5 | 5 | 5 | 5 | 5 |
|  | 7 (rev) | 4.91 | 0.34 | 3 | 5 | 5 | 5 | 5 | 5 |
|  | 8 (rev) | 4.96 | 0.26 | 3 | 5 | 5 | 5 | 5 | 5 |
|  | 9 (rev) | 4.95 | 0.37 | 2 | 5 | 5 | 5 | 5 | 5 |
| Overall Global Rating | 4.78 | | 0.50 | 2 | 5 | 5 | 5 | 5 | 5 |

| Table S3. Item response correlations for the PROMIS Medication Adherence Scale at time points T1 and T2 (3- and 6-months post study enrollment) | | | | | | | | | | |
| --- | --- | --- | --- | --- | --- | --- | --- | --- | --- | --- |
| **T1** |  | **1** | **2** | **3** | **4** | **5** | **6** | **7** | **8** | **9** |
|  | **1** | 1 |  |  |  |  |  |  |  |  |
|  | **2** | 0.922 | 1 |  |  |  |  |  |  |  |
|  | **3** | 0.961 | 0.942 | 1 |  |  |  |  |  |  |
|  | **4** | 0.867 | 0.915 | 0.919 | 1 |  |  |  |  |  |
|  | **5** | 0.193 | 0.236 | 0.249 | 0.249 | 1 |  |  |  |  |
|  | **6** | -.021 | 0.021 | 0.000 | 0.042 | 0.642 | 1 |  |  |  |
|  | **7** | -.028 | 0.003 | 0.012 | 0.067 | 0.156 | 0.041 | 1 |  |  |
|  | **8** | 0.113 | 0.102 | 0.246 | 0.216 | 0.499 | 0.206 | 0.315 | 1 |  |
|  | **9** | 0.022 | 0.011 | 0.079 | 0.054 | 0.176 | 0.040 | 0.425 | 0.437 | 1 |
| **T2** |  | **1** | **2** | **3** | **4** | **5** | **6** | **7** | **8** | **9** |
|  | **1** | 1 |  |  |  |  |  |  |  |  |
|  | **2** | 0.832 | 1 |  |  |  |  |  |  |  |
|  | **3** | 0.881 | 0.951 | 1 |  |  |  |  |  |  |
|  | **4** | 0.755 | 0.832 | 0.846 | 1 |  |  |  |  |  |
|  | **5** | 0.158 | 0.168 | 0.182 | 0.215 | 1 |  |  |  |  |
|  | **6** | 0.298 | 0.222 | 0.247 | 0.286 | 0.394 | 1 |  |  |  |
|  | **7** | 0.128 | 0.138 | 0.230 | 0.134 | 0.199 | 0.202 | 1 |  |  |
|  | **8** | 0.091 | 0.098 | 0.159 | 0.230 | 0.668 | 0.072 | 0.269 | 1 |  |
|  | **9** | 0.101 | 0.109 | 0.149 | 0.232 | 0.678 | 0.035 | 0.179 | 0.990 | 1 |
